# Case report: Creutzfeldt-Jakob disease: a case that initiated with the onset of obsessive-compulsive state

**DOI:** 10.3389/fneur.2023.1227566

**Published:** 2023-07-18

**Authors:** Baizhu Li, Shan Wang, Shiyu Han, Ning Hu, Xiuli Shang

**Affiliations:** The First Affiliated Hospital of China Medical University, Shenyang, China

**Keywords:** obsessive-compulsive disorder, compulsive state, Creutzfeldt-Jakob disease, early diagnosis, case report, CJD

## Abstract

**Background:**

Obsessive-compulsive disorder (OCD) is a common reason for patients to seek symptomatic treatment in psychiatric departments, which makes it challenging to consider underlying organic nervous system diseases. However, Creutzfeldt-Jakob disease (CJD) can present with atypical symptoms, sometimes even as initial symptoms, leading to misdiagnosis or missed diagnosis. Lumbar puncture and brain DWI are important diagnostic methods for CJD, and the detection of 1,433 protein can be performed to confirm the diagnosis.

**Case presentation:**

We present the case of a 63-year-old woman who was initially diagnosed with obsessive-compulsive disorder in 2022. Despite seven months of symptomatic treatment, her symptoms did not improve. She also developed symptoms of altered consciousness, such as upper limb tremors and mutism. Based on brain DWI and positive results from the detection of 1,433 protein, she was ultimately diagnosed with CJD.

**Conclusion:**

Creutzfeldt-Jakob disease (CJD) can manifest initially as obsessive-compulsive disorder (OCD) with atypical symptoms, making it prone to misdiagnosis. Therefore, it is crucial to conduct further investigations, including lumbar puncture and imaging, to exclude organic nervous system diseases before initiating symptomatic treatment for psychiatric disorders. This approach can facilitate early diagnosis of CJD and other potential organic neurological diseases.

## Introduction

Creutzfeldt-Jakob disease (CJD) is a progressive, degenerative, and ultimately fatal disease affecting the central nervous system. It is characterized by the abnormal accumulation of prion protein, leading to various neurological symptoms. The disease presents with progressive dementia, myoclonus (involuntary muscle jerks or twitches), and manifestations of cerebellar, pyramidal, and extrapyramidal dysfunction (1). It has been reported that psychiatric symptoms, including depression, can accompany Creutzfeldt-Jakob disease (CJD) and may even present as the initial signs of the disease. Alongside the neurological symptoms, individuals with CJD may experience changes in mood, behavior, and cognition, which can include depression. Therefore, it is important to consider psychiatric manifestations as potential indicators of CJD, especially when evaluating patients with unexplained psychiatric symptoms in conjunction with neurological abnormalities (2). Recently, it has been observed that obsessive-compulsive disorder (OCD) can also serve as an early manifestation of Creutzfeldt-Jakob disease (CJD). In this report, we present a case where a patient was initially diagnosed with OCD by a psychiatrist but later diagnosed with CJD. The patient exhibited progressive dementia, myoclonus, immobility mutism, and other characteristic manifestations of CJD. The diagnosis of CJD was confirmed through additional investigations, including lumbar puncture and brain DWI examination. This case highlights the importance of considering CJD as a potential underlying cause when encountering patients with OCD symptoms accompanied by neurological deterioration (3). Compulsive behavior or psychological symptoms can indeed be part of the clinical manifestations of Creutzfeldt-Jakob disease (CJD). Recognizing these symptoms and utilizing additional diagnostic tools such as lumbar puncture and imaging can enhance our understanding of organic nervous system diseases in patients presenting with such mental states. These auxiliary means contribute to early diagnosis and prompt treatment, ultimately improving clinical outcomes. Early identification of CJD and other organic neurological conditions in individuals displaying compulsive behavior or psychological symptoms is crucial for appropriate management and support.

## Patient and method

This article reports a patient who was initially diagnosed with obsessive-compulsive disorder but was finally diagnosed with Creutzfeldt-Jakob disease after 1 year of follow-up observation.

This is a retrospective study of the patient characteristics and treatment outcomes, ethical committee approval was not required for this study, written informed consent of all patients have been obtained.

## Case

A 63-year-old woman developed stereotypical behavior in November 2022 without any apparent cause. She engaged in repetitive actions such as washing clothes for an entire day, repeatedly washing the same batch of clothes in the washing machine, or continuously wiping the floor or dust. She displayed heightened focus on personal and household hygiene, frequently washing her hands, while neglecting other household tasks and becoming lazier. However, her speech and self-care abilities remained normal.

On January 26, 2023, when she learned about her father's death in a car accident, her family noticed an increase in repetitive behaviors. These included repeatedly wiping parts of the floor and excessively washing her hands from morning till night. The symptoms worsened over the course of a week. Subsequently, she settled into a regular routine of compulsively washing her hands and wiping the floor. As a result, she was diagnosed with obsessive-compulsive disorder (OCD) at another hospital and received symptomatic medication for 2 weeks. However, the symptoms did not improve.

On March 7, 2023, she was admitted to the psychiatric department of my hospital, where she received a diagnosis of obsessive-compulsive disorder and underwent medication-based treatment. During the treatment, she frequently noticed her hand assuming a fixed position, such as being in a booth or elbow flexion, and experienced involuntary tremors in her left hand, although her muscle strength remained normal. The patient's ability to express herself through speech decreased, and she could only produce single words. She often did not answer phone calls, spent less time awake, experienced difficulty walking compared to before, had frequent trembling in her right hand, cried frequently, and exhibited significant mood fluctuations. However, the following tests and examinations did not reveal any notable abnormalities: rheumatic diseases, coagulation, blood routine, liver and kidney function, syphilis, brucella, AIDS, hepatitis C virus, hepatitis B virus, troponin, ion, erythrocyte sedimentation rate, thyroid function, procalcitonin, tumor markers, anticardiolipin antibodies, electrocardiogram, electroencephalogram, brain CT, MR plain scan +C, and related cervical, thoracic, and lumbar vertebrae MR. In addition to the above, I did a whole exome analysis on the patient, and did not find any genetic variant, which may be responsible for the disease.

**Figure 1:**
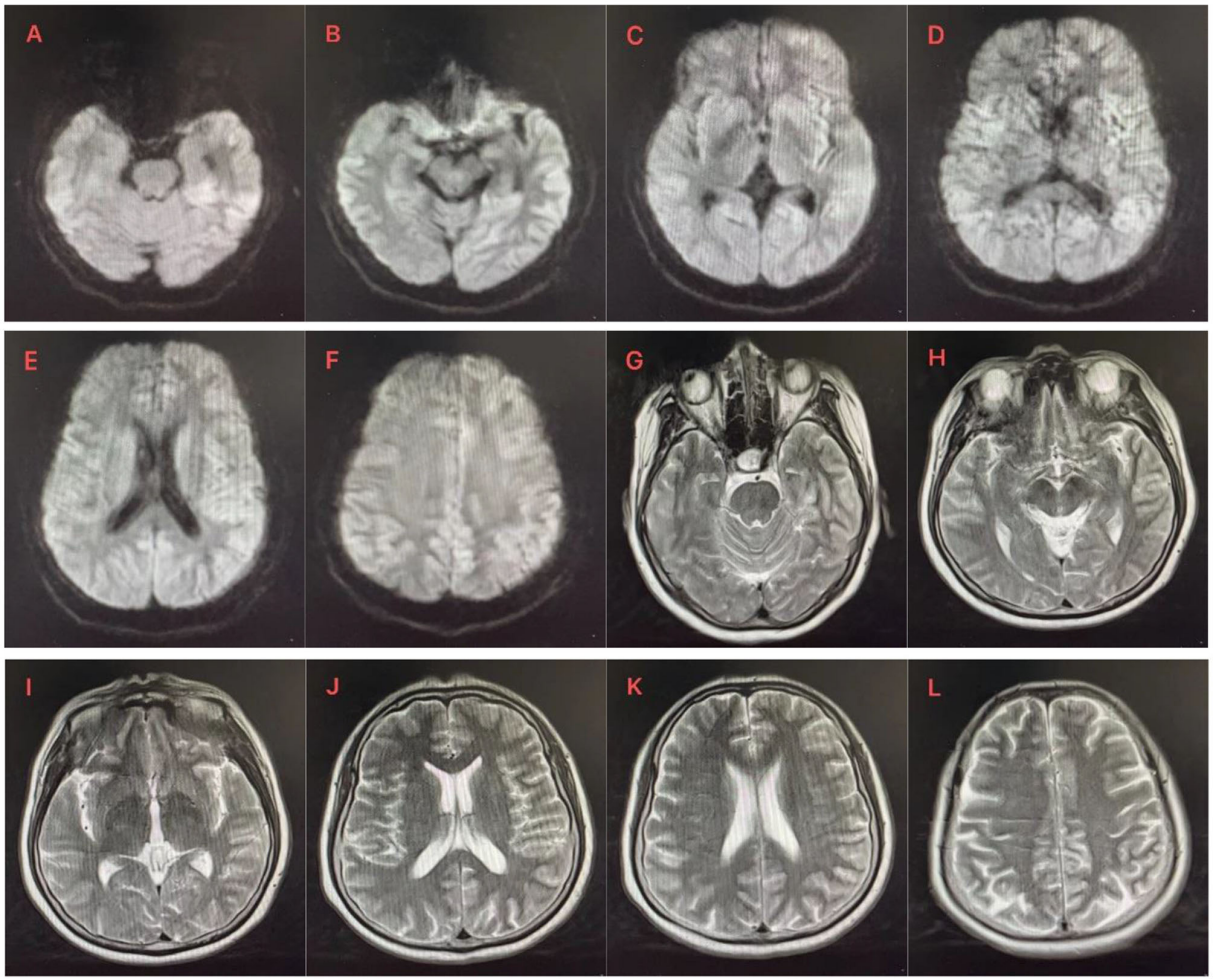
**(A–F)** DWI showed asymmetric cortical hyperintensity in the bilateral caudate nucleus, bilateral cingulate gyrus and frontoparietal, temporal and occipital gyrus, especially in the left. **(G–L)** Small patchy long T2 signal shadow was observed next to the posterior horn of the left lateral ventricle. There was no abnormal signal shadow in the remaining bilateral cerebral hemispheres, cerebellum, and brain stem, no abnormal morphological structure was found, the ventricular system was equal and symmetrical, the sulci cistern fissure was not significantly widened, and the midline structure was centered.

**Figure 2:**
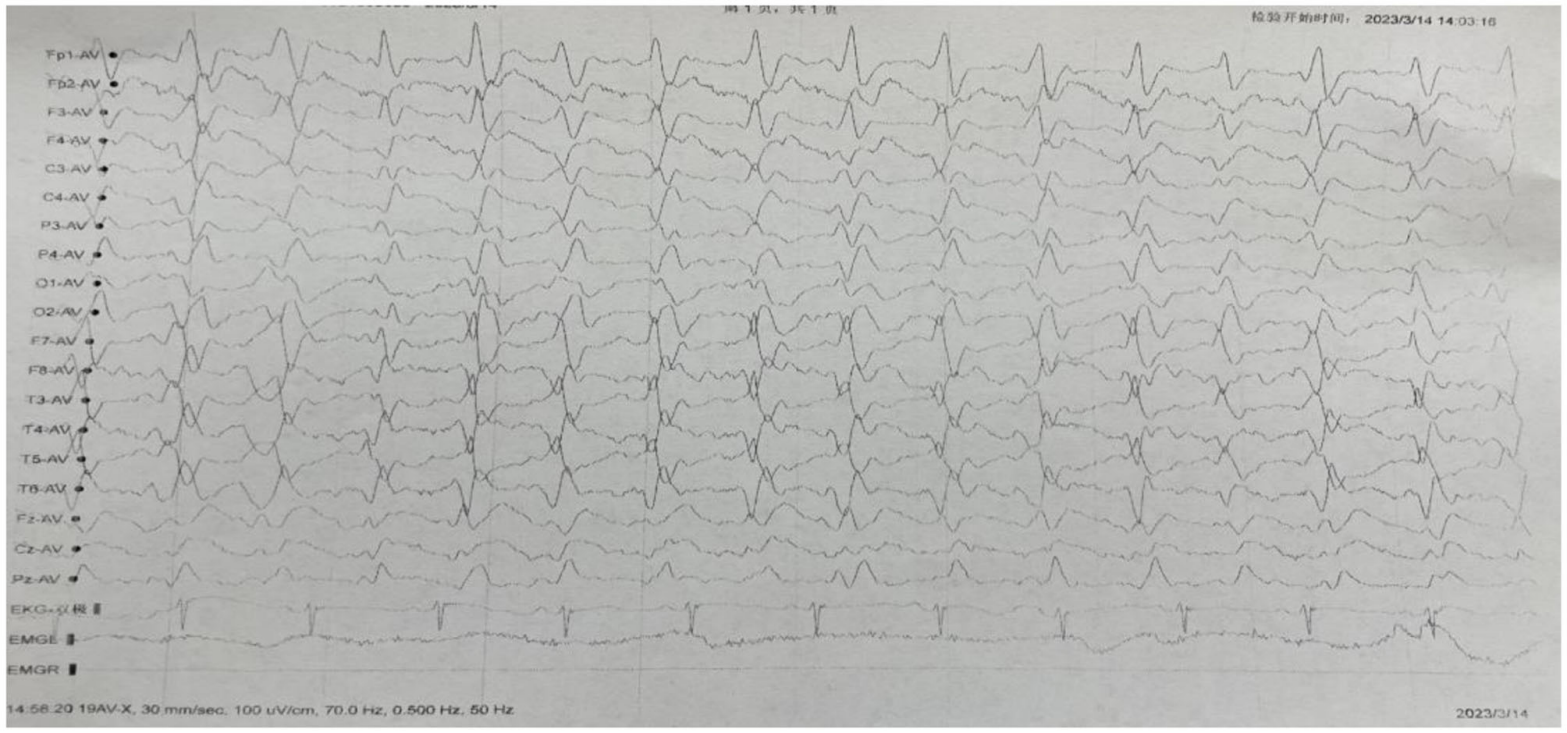
Electroencephalogram (EEG) showed that the basic rhythm disappeared, periodic three-phase waves appeared in all leads, with an inter-wave interval of 0.5–0.7s.

After consultation with the neurology department on April 23, 2023, and ruling out the aforementioned symptoms, laboratory test results, and any obvious abnormalities in the examination results, dementia-related causes were excluded. When the patient's medical history and family genetic history were investigated, the patient's family members denied any relevant medical history or neurological symptoms within the family (Figure 1). Further examination of diffusion-weighted imaging (DWI) results revealed multiple abnormal diffusion-restricted signal changes in the brain with cortical and subcortical distribution, indicating a high possibility of infectious lesions and Creutzfeldt-Jakob disease (CJD) (Figure 2). A reexamination of the video electroencephalogram (VEEG) demonstrated the disappearance of the basic rhythm and the appearance of periodic triphasic waves in all leads. During monitoring, the patient exhibited increased muscle tension in the right upper limb with elbow flexion, and frequent rhythmic convulsions occurred in the distal upper limb. The EEG results showed widespread and severe abnormalities. The 1,433 protein test yielded a positive result, confirming the diagnosis of Creutzfeldt-Jakob disease. Following her discharge, the patient passed away in May 2023 during the follow-up period.

The temporal and occipital leads were predominant. A small amount of a waves (10–40μv, 8–9c/s), β waves (5–20μv, 18–24c/s) and δθ waves (10–100μv, 2–7c/s) were mixed. Open and closed eyes test: a wave was not suppressed. Response to flash stimulation: same as background figure. Sphenoid electrode: No seizure wave was observed. Clinical seizure: during the monitoring period, the patient's right upper limb muscle tension was increased in the elbow flexion position, and the distal end of both upper limbs frequently had rhythmic twitches. The corresponding electroencephalogram showed that the electromyogram showed motor unit potential, and the other leads were the same as the background map.

Video electroencephalogram results: extensive severe abnormal electroencephalogram.

## Discussion

Creutzfeldt-Jakob disease (CJD) is the most common prion disease in humans, with an annual incidence of approximately 1 case per million people (4). The typical onset age of CJD is between 45 and 75 years old, with an average age of onset at 60 years old (5, 6). Apart from dementia, the most common manifestations of CJD are pyramidal, extrapyramidal, and cerebral manifestations, and myoclonus. The typical manifestations of the disease, namely dementia, myoclonic convulsions, cranial MR, and EEG changes, may not be observed in 25% of patients, leading to difficulties in early diagnosis. There are three main categories of prion diseases (7), called sporadic, hereditary and acquired, of which 85% to 95% are sporadic (no family history or source of infection can be identified) and 5% are familial. In addition to typical symptoms, patients can also start with neuropsychiatric symptoms. Psychiatric symptoms, such as depression and personality changes, have been defined in one third of cases early in the illness (1), and are the initial symptoms in 10% of cases. Delusions and auditory hallucinations have been reported in patients with major depression for about 6 months, and ataxia and myoclonus have developed 7 months after the onset of these symptoms (1). It has also been shown that symptoms such as visual hallucinations are observed after depressive symptoms in 15% of patients with sporadic CJD (8, 9). Except for the difference in the first symptom, the onset time of the other symptoms was similar to that of this case, in which CJD first manifested as obsessive-compulsive disorder, repeatedly wiping the floor and ashes, and paying great attention to hygiene, which was extremely difficult to distinguish from obsessive-compulsive disorder patients in terms of clinical manifestations (10). There was no significant change in electroencephalogram and no response to symptomatic treatment. Within 6 months, the patient developed visual hallucinations, auditory hallucinations, myoclonus and other symptoms, which were consistent with the early symptoms of CJD patients. Moreover, the duration of clinical psychiatric symptoms of the disease is shorter (1) and the age of onset is later. Reexamination of electroencephalogram, brain DWI and positive delivery 1, 433 protein (11) supported the diagnosis of sporadic CJD.

These features on EEG are known to occur in the middle or late stages of CJD, but are not specific for CJD in the early stages. Therefore, the EEG of patients with suspected CJD should be reexamined many times. In this case, there was no obvious abnormality in the initial EEG, but periodic three-phase waves appeared in all leads of the EEG in the later stage, and the video EEG showed extensive and severe abnormal EEG. This is characteristic of sporadic CJD and can be observed in two thirds of CJD patients.

Brain MRI is an important examination for the diagnosis of CJD. MRI findings may be normal in 23% of patients with sporadic CJD (12). It has been reported that cranial DWI can be used even in the early stages of CJD, and it is an important modality for early diagnosis (13). In our patient, the first brain plain MR Scan showed no obvious abnormality in DWI, and then the brain DWI showed multiple abnormal diffusion limited signal changes in the brain, with cortical and subcortical distribution, considering infectious lesions and Creutzfeldt-Jakob disease.

In patients with CJD, CSF examination may be positive for 14–3-3 (14). The sensitivity and specificity of 14–3-3 for CJD were 94% and 84%, respectively (15). Therefore, a positive 14–3-3 result in CSF suggests a certain diagnosis in patients with sporadic CJD. In the present case, 14–3-3 was found in the patient's CSF.

## Conclusion

In this report, we present a case of Creutzfeldt-Jakob disease (CJD) where the initial symptom was obsessive-compulsive disorder (OCD), and the patient was followed up for a period of 7 months. The challenging aspect of this case was that the patient's initial symptoms were purely psychiatric, making it difficult to differentiate them from common psychiatric disorders. By highlighting this case, we aim to raise awareness among clinicians about the importance of recognizing psychiatric symptoms, such as OCD, as potential early signs of CJD and other organic nervous system diseases. CJD should be considered as a differential diagnosis in patients who present with psychiatric symptoms, personality changes, and focal neurological symptoms. This case is particularly significant as it demonstrates that neurological symptoms can manifest after the onset of psychiatric symptoms in CJD.

Therefore, when evaluating patients with psychiatric symptoms, it is crucial to employ methods like lumbar puncture, imaging, and other diagnostic approaches to exclude organic nervous system diseases before initiating symptomatic treatment for psychiatric conditions. This approach can help avoid misdiagnosis of a progressive disease like CJD and differentiate it from other potentially treatable causes.

## Data availability statement

The original contributions presented in the study are included in the article/Supplementary material, further inquiries can be directed to the corresponding author.

## Ethics statement

Ethical review and approval was not required for the study on human participants in accordance with the local legislation and institutional requirements. The patients/participants provided their written informed consent to participate in this study. Written informed consent was obtained from the individual(s) for the publication of any potentially identifiable images or data included in this article.

## Author contributions

BL wrote the manuscript. BL and XS revised the manuscript. All authors contributed to the follow-up, information collection, and approved the submitted version.
